# Genomic prediction of antimicrobial resistance in priority healthcare pathogens using Staramr

**DOI:** 10.1128/spectrum.01652-26

**Published:** 2026-10-06

**Authors:** MacKenzie A. P. Wilke, Laura F. Mataseje, Nicole Lerminiaux, Melissa McCracken, Romaine Edirmanasinghe, Melanie Baxter, Ken Fakharuddin, Gurman Grewal, Xiao Rui Li, Eric Marnier, Aaron Petkau, Michael Mulvey, George Zhanel, George R. Golding, Amrita Bharat

**Affiliations:** 1 National Microbiology Laboratory, Public Health Agency of Canada 85072 , Winnipeg, Manitoba, Canada; 2 Department of Medical Microbiology and Infectious Diseases, University of Manitoba 574854, Winnipeg, Manitoba, Canada; University of Manitoba, Winnipeg, Manitoba, Canada

**Keywords:** carbapenemase, *Escherichia coli*, *Klebsiella pneumoniae*, vancomycin-resistant *Enterococcus*, methicillin-resistant *Staphylococcus aureus*, antimicrobial resistance prediction, Staramr, ResFinder, PointFinder

## Abstract

Treatment of healthcare-associated infections is becoming increasingly challenging due to rising antimicrobial resistance (AMR). As clinical microbiology and public health continue to transition toward whole-genome sequencing (WGS) for outbreak detection, diagnostics, and surveillance, the ability to accurately predict antimicrobial resistance from genomic sequence data will become crucial. In this study, *in silico* antimicrobial resistance prediction with the Staramr tool was refined from whole-genome sequencing data for four top-priority healthcare-associated bacteria: carbapenemase-producing *Escherichia coli*, carbapenemase-producing *Klebsiella pneumoniae*, methicillin-resistant *Staphylococcus aureus*, and vancomycin-resistant *Enterococcus faecium*. Isolates (*n* = 4,876) were collected in Canada within two national surveillance programs: the Canadian Nosocomial Infection Surveillance Program and the Canadian Ward Study. Broth microdilution was used as the gold standard to evaluate the performance of Staramr. The average sensitivity, specificity, and concordance of categorical antimicrobial resistance prediction compared to broth microdilution were 90.8%, 90.1%, and 92.4% for *E. coli* (*n* = 1,095); 87.7%, 91.7%, and 90.6% for *K. pneumoniae* (*n* = 687); 95.5%, 98.9%, and 96.3% for methicillin-resistant *S. aureus* (*n* = 2,150); and 95.4%, 97.9%, and 97.2% for vancomycin-resistant *Enterococcus faecium* (*n* = 944). Additionally, this internal tool refinement identified areas of improvement for antimicrobial resistance prediction. The average sensitivity, specificity, and concordance of antimicrobial resistance prediction were high (>90%) for most combinations of antimicrobials and species that were evaluated. While AMR prediction from WGS was very promising, it did not correlate well with current Clinical and Laboratory Standards Institute breakpoints for non-susceptibility for some antimicrobial-organism combinations. As *in silico* prediction of antimicrobial resistance has been improved in this study through tool refinement, it shows future promise for molecular methods to complement phenotypic antimicrobial susceptibility testing. This will allow for more precise tracking of the molecular epidemiology of resistance and cost savings when isolates are being sequenced for strain typing.

**IMPORTANCE:**

*In silico* prediction of antimicrobial non-susceptibility in priority healthcare pathogens is becoming an important area of study due to the rising rates of resistance and the transition to whole-genome sequencing. To evaluate whether Staramr, an antimicrobial resistance (AMR) prediction tool, can reliably detect non-susceptibility in four top-priority healthcare-associated bacteria, we performed a validation study on a large Canadian clinical data set, which included carbapenemase-producing *Escherichia coli* and *Klebsiella pneumoniae*, methicillin-resistant *Staphylococcus aureus,* and vancomycin-resistant *Enterococcus*. Compared to traditional antimicrobial susceptibility testing, we found that Staramr was reliable for predicting non-susceptibility for most combinations of antimicrobials and organisms; however, some combinations require additional data on genetic mechanisms of resistance for accurate prediction. Additionally, this study identified areas for refinement for current drug-gene interpretation keys. Thus, this tool refinement study advances the use of genotypic methods of AMR prediction for high-priority healthcare pathogens.

## INTRODUCTION

Antimicrobial resistance (AMR) is a critical global health and economic threat. Hundreds of thousands of deaths occur each year due to AMR infections, and projections estimate that 1.9–10 million deaths may occur annually by 2050 (1–4). By 2050, the global economic burden of AMR is projected to approach 2 trillion USD (3, 5, 6).

The highest priority healthcare-associated gram-negative pathogens are carbapenemase-resistant organisms (CROs), as these organisms are resistant to carbapenems and are often co-resistant to many other antimicrobial classes, leaving few effective treatment options (5, 7). *Escherichia coli* and *Klebsiella pneumoniae* are the two most common CROs (8). Carbapenemase-producing CROs are especially concerning because they often harbor mobilizable carbapenemases and plasmids that can transfer within *Enterobacterales*. For gram-positive bacteria, the highest priority organisms in healthcare settings are vancomycin-resistant *Enterococcus* (VRE) and methicillin-resistant *Staphylococcus aureus* (MRSA) (5, 7). Surveillance of these top-priority pathogens in Canada is monitored by the Canadian Nosocomial Infection Surveillance Program (CNISP) (8) and the Canadian Ward Study (CANWARD) led by the Canadian Antimicrobial Resistance Alliance (9, 10).

*In silico* AMR prediction studies have been increasingly described, and promising results have been observed. Staramr is a user-friendly *in silico* AMR prediction tool developed by the Public Health Agency of Canada (PHAC) for *Salmonella enterica*. The program produces quality control assembly metrics and applies BLAST-based sequence analysis to identify AMR-associated genes and mutations in bacterial genome contigs (11). Staramr incorporates results from the bacterial sequencing typing database from PubMLST and databases from the Center for Genomic Epidemiology (CGE), including ResFinder for acquired resistance genes (12), PointFinder for species-specific chromosomal mutations (13), and PlasmidFinder for plasmid identification (11, 14–18). It then converts the detected genotype to a predicted phenotype based on either the gene-drug key from the CGE or a gene-drug key developed by the US Centers for Disease Control (CDC) for enteric bacteria (https://github.com/phac-nml/staramr/tree/development/staramr/databases/resistance/data). Users can choose whether to analyze predicted antimicrobial phenotypes from the *Salmonella*-focused CDC key or the more general clinically focused CGE key. Both interpretation columns list antimicrobials for which the isolate is predicted to be resistant based on its genotype.

Here, we performed an internal tool refinement of Staramr for AMR prediction in four top-priority healthcare-associated pathogens: carbapenemase-producing *E. coli*, carbapenemase-producing *K. pneumoniae*, MRSA, and VRE using a robust sample set of over 4,876 human clinical isolates.

## MATERIALS AND METHODS

Isolates for this study were collected by CNISP (8), including carbapenemase-producing *E. coli*, carbapenemase-producing *K. pneumoniae*, MRSA, and VRE (*Enterococcus faecium* only). We also included carbapenem-susceptible isolates from CANWARD surveillance (9) for *E. coli* and *K. pneumoniae*. The refinement exercise includes isolates for which both minimum inhibitory concentrations (MICs) from broth microdilution and whole-genome sequencing (WGS) data were available. This included 1,095 *E. coli* isolates from CNISP (*n* = 325) and CANWARD (*n* = 770), 687 *K. pneumoniae* isolates from CNISP (*n* = 384) and CANWARD (*n* = 303), 2,150 MRSA from CNISP, and 944 VRE from CNISP. Phenotypic antimicrobial susceptibility testing by broth microdilution was carried out according to the Clinical and Laboratory Standards Institute (CLSI) (19) as previously described (11, 20). MIC categorizations were interpreted according to clinical guidelines (19), as detailed in Table 1. Where available, CLSI breakpoints were used. For tigecycline, the US Food and Drug Administration (FDA) breakpoints (21) were used for *E. coli*, *K. pneumoniae,* and MRSA, as no interpretation was available in CLSI. For *Enterococcus* and tigecycline, there was no FDA breakpoint; therefore, we used the EUCAST breakpoint (22). For *E. coli* and *K. pneumoniae,* we evaluated the following 13 antimicrobials: cefepime, ceftazidime, ceftriaxone, ciprofloxacin, colistin, doxycycline, ertapenem, gentamicin, meropenem, piperacillin-tazobactam, tigecycline, tobramycin, and trimethoprim-sulfamethoxazole. For MRSA and VRE, we evaluated the following antimicrobials in both gram-positive organisms: chloramphenicol, ciprofloxacin, daptomycin, erythromycin, linezolid, penicillin, quinupristin-dalfopristin, rifampicin, tetracycline, tigecycline, and vancomycin. Additionally, in VRE, ampicillin was tested, and in MRSA, clindamycin and trimethoprim-sulfamethoxazole were tested.

**TABLE 1:** Non-susceptibility clinical breakpoints used for validation of AMR prediction

*E. coli* and *K. pneumoniae* antimicrobials (non-susceptibility breakpoint [mg/L])^*a*^	MRSA antimicrobials (non-susceptibility breakpoint [mg/L])	VRE antimicrobials (non-susceptibility breakpoint [mg/L])
Cefepime (≥4)	Chloramphenicol (≥16)	Ampicillin (>8)
Ceftazidime (≥8)	Ciprofloxacin (≥2)	Chloramphenicol (≥16)
Ceftriaxone (≥2)	Clindamycin^*c*^	Ciprofloxacin (≥2)
Ciprofloxacin (≥0.5)	Daptomycin (>1)	Daptomycin (>4)
Colistin (≥4)	Erythromycin (≥1)	Erythromycin (≥1)
Doxycycline (≥8)	Linezolid (>4)	Linezolid (≥4)
Ertapenem (≥1)	Penicillin (>0.12)	Penicillin (>8)
Gentamicin (≥4)	Quinupristin-dalfopristin (≥2)	Tetracycline (≥8)
Meropenem (≥2)	Rifampicin (≥2)	Tigecycline^*d*^ (>0.5)
Piperacillin-tazobactam (≥16/4)	Tetracycline (≥8)	Vancomycin (≥8)
Tigecycline^*b*^ (≥4)	Tigecycline^*b*^ (>0.5)	
Tobramycin (≥4)	Trimethoprim-sulfamethoxazole (>2/38)	
Trimethoprim-sulfamethoxazole (>2/38)	Vancomycin (≥4)	

^*a*^ Breakpoints are based on Clinical and Laboratory Standards Institute (M100-ED35:2025) when available, and exceptions are noted.

^*b*^ Uses Food and Drug Administration guidelines.

^*c*^ Utilizing an in-house clinical breakpoint calculation that considers erythromycin, clindamycin, Dtest1, and Dtest2 values.

^*d*^ Uses European Committee on Antimicrobial Susceptibility Testing 2026 guidelines (v.16.0).

DNA extraction was performed using the DNeasy 96 Blood and Tissue Kit (QIAGEN, Hilden, Germany) from 2010 to 2021 and the Mag-Bind Universal Pathogen DNA 96 Kit (Omega Bio-Tek, Norcross, Georgia, USA) from 2022 to 2024 (23). In addition, we supplemented the kits with bead-beating for all organisms, an additional lysostaphin treatment for *S. aureus*, and lysozyme for *E. faecium*. Illumina sequencing was performed on all isolates, and a minimum of 50× average coverage across the sequenced regions was applied. Shovill v1.1.0 (24) was used to assemble Illumina fastq files into fasta files. Genome completeness was analyzed using CheckM2 (25) for all organisms, and the average completeness was 99.9% for *E. coli*, *K. pneumoniae*, MRSA, and VRE. Since Staramr includes quality control of genome assembly inputs, we also applied the following quality filters: genome size 2.0–4.0 Mb for MRSA and VRE and 4.0–6.7 Mb for *E. coli* and *K. pneumoniae; N*_50_ >3,000 bp for MRSA and VRE and >10,000 bp for *E. coli* and *K. pneumoniae*. For AMR prediction, we used Staramr v0.11.0 with search parameters: 90% identity for BLAST, 60% length overlap for ResFinder BLAST, and 95% length overlap for PointFinder BLAST. Staramr only reports the top BLAST hit among similar alleles as detailed in the source code (https://github.com/phac-nml/staramr/blob/02f4e7056b2cb21471b0063f6ebde9ce6d70c809/staramr/blast/results/BlastResultsParser.py#L136-L140). For example, if an isolate contained *bla_NDM-1_*, similar alleles like *bla_NDM-5_* would not be reported. We used the CGE key output column of Staramr for determining WGS non-susceptibility; drug-gene versions used were v.07423 for ResFinder and v.070623 for PointFinder.

We evaluated the diagnostic sensitivity and specificity of categorical AMR prediction by Staramr (susceptible vs non-susceptible [intermediate or resistant]) by treating phenotypic MIC data as the gold standard. This categorization is more closely aligned with traditional antibiograms. We used a confusion matrix approach by assigning binary identifiers (1 = non-susceptible and 0 = susceptible) to phenotypic and genotypic results and comparing them in a two-by-two table. Figure S1 details the definitions of true negatives, true positives, false negatives, and false positives, as well as the formulas used to calculate sensitivity, specificity, and concordance. The following definitions are used: a true negative is when both the phenotype and genotype results are susceptible; a true positive is when both are non-susceptible; a false negative is when the phenotype is non-susceptible and the genotype is susceptible (failure to detect non-susceptibility); and a false positive is when the phenotype is susceptible and the genotype is non-susceptible (overcalling non-susceptibility). Sensitivity was calculated as $\left[\frac{true\;positives}{\left(true\;positives+false\;negatives\right)}\right]\times 100$, specificity was calculated as $\left[\frac{true\;negatives}{\left(true\;negatives+false\;positives\right)}\right]\times 100$, and concordance as $\left[\frac{sample\;size-\left(false\;negatives+false\;positives\right)}{sample\;size}\right]\times 100$. Major error rate was calculated as $(\frac{false\;positive}{false\;positives+true\;negatives})\times 100$, and very major error rate was calculated as $\left(\frac{false\;negatives}{false\;negatives+true\;positives}\right)\times 100$.

For gram-negative and gram-positive organisms in this study, we used CLSI non-susceptibility breakpoints as outlined in Table 1, except for tigecycline, as previously mentioned, and clindamycin for MRSA, which had an in-house interpretation. When less than 1% of isolates were susceptible or non-susceptible to an antimicrobial according to broth microdilution, it was noted that there were insufficient data to assess sensitivity or specificity, respectively. Additionally, when calculating the very major error, when there were no non-susceptible isolates (no true positives or false negatives), an “NA” value was recorded. All data cleaning, analysis, and visualization steps were performed in R Statistical Software (26) (v4.4.3) using the open-source packages dplyr, ggplot2, ggtext, openxlsx, janitor, data.table, caret, and tidyr.

As we were focusing on internal tool development and refinement of Staramr for these top-priority healthcare pathogens, troubleshooting was performed for low predictive statistics. When sensitivity for a particular antimicrobial was low, we cross-checked false negatives using other AMR prediction software such as AMRFinderPlus (27) and CARD (28) to identify genetic determinants that were supported by our MIC data and therefore should be incorporated into the next Staramr drug-gene key. For example, in gram-negative bacteria, colistin resistance prediction was greatly improved when using results from both Staramr and AMRFinderPlus or if using a version of the Staramr key with the additional *pmrB* allele mutations: E123D, P94S, and C84Y.

## RESULTS

We evaluated the performance of AMR prediction in 4,876 human clinical isolates of four priority healthcare-associated bacteria: *E. coli* (*n* = 1,095), *K. pneumoniae* (*n* = 687), MRSA (*n* = 2,150), and VRE (*n* = 944) through internal Staramr tool refinement. The *E. coli* and *K. pneumoniae* data sets were biased toward carbapenemase producers. Categorical results (non-susceptible or susceptible) from broth microdilution were treated as the gold standard. Tables S1A and B outline the percentage of non-susceptibility according to MIC and WGS results for each antimicrobial.

### E. coli

We evaluated AMR prediction in 1,095 *E. coli* isolates for 13 antimicrobials using non-susceptibility clinical MIC breakpoints (Table 2). A total of 325 isolates were from the CNISP carbapenemase-producing *Enterobacterales* (CPE) surveillance program, and 770 were non-CPE isolates from the CANWARD surveillance program. The data set was biased toward carbapenemase-producing *E. coli* since our objective was to validate and improve AMR prediction in CPEs; 17.2% and 20.1% of *E. coli* were non-susceptible to meropenem based on phenotypic and genotypic data, respectively (Table S1A).

**TABLE 2:** *In silico* antimicrobial resistance predictive statistics for *Escherichia coli* using Staramr and non-susceptible clinical breakpoints (*n* = 1,095)

	Non-susceptible		Susceptible		Sensitivity (%)	Specificity (%)	Concordance (%)	ME^*b*^ (%)	VME^*b*^ (%)	CLSI^*c*^ tier of drug
Antimicrobial^*a*^	TP^*b*^	FN^*b*^	TN^*b*^	FP^*b*^						
CEF	596	21	339	139	96.6	70.9	85.4	29.1	3.4	2
CAZ	612	9	335	139	98.6	70.7	86.5	29.3	1.5	4
CRO	627	128	333	7	83.1	97.9	87.7	2.1	17.0	1
CIP	750	2	257	86	99.7	74.9	92.0	25.1	0.3	1
COL	2	6	1,086	1	NA^*d*^	99.9	99.4	0.1	75.0	
DOX	588	23	465	19	96.2	96.0	96.2	3.9	3.8	
ERT	186	103	789	17	64.4	97.9	89.0	2.1	35.6	2
GEN	382	12	693	8	97.0	98.9	98.2	1.1	3.1	1
MER	158	30	845	62	84.0	93.2	91.6	6.8	16.0	2
TZP	341	62	551	141	84.6	79.6	81.5	20.4	15.4	1
TGC	0	0	1,094	1	NA^*d*^	99.9	99.9	0.1	NA^*e*^	
TOB	482	14	571	28	97.1	95.3	96.2	4.7	2.8	2
SXT	629	14	436	16	97.8	96.5	97.3	3.5	2.2	1
Average					90.8	90.1	92.4			

^*a*^ CEF, cefepime; CAZ, ceftazidime; CRO, ceftriaxone; CIP, ciprofloxacin; COL, colistin; DOX, doxycycline; ERT, ertapenem; GEN, gentamicin; MER, meropenem; TZP, piperacillin-tazobactam; TGC, tigecycline; TOB, tobramycin; and SXT, trimethoprim-sulfamethoxazole.

^*b*^ TP, true positive; FN, false negative; TN, true negative; FP, false positive; ME, major error; and VME, very major error.

^*c*^ CLSI, Clinical and Laboratory Standards Institute M100-ED35:2025.

^*d*^ Less than 1% of *E. coli* isolates were non-susceptible to colistin or tigecycline; therefore, sensitivity could not be assessed for these antimicrobials.

^*e*^ Unable to calculate as true positives and false negatives are equal to 0 (no non-susceptible isolates).

The concordance between broth microdilution and AMR prediction of resistance for 13 antimicrobials in *E. coli* was on average 92.4% (range, 81.5%–99.9%) (Table 2). The sensitivity of prediction of AMR resistance averaged 90.8% (range, 64.4%–99.7%) and the specificity averaged 90.1% (70.7%–99.9%). Sensitivities were ≥85% for eight antimicrobials (cefepime, ceftazidime, ciprofloxacin, doxycycline, gentamicin, tobramycin, and trimethoprim-sulfamethoxazole) and <85% for piperacillin-tazobactam (84.6%), meropenem (84.0%), ceftriaxone (83.1%), and ertapenem (64.4%). Colistin and tigecycline had less than 1% of isolates with non-susceptible MIC values, and therefore, no sensitivity value could be calculated.

All specificities were ≥85% except for piperacillin-tazobactam (79.6%), ciprofloxacin (74.9%), cefepime (70.9%), and ceftazidime (70.7%). Concordance was ≥85% for all antimicrobials except for piperacillin-tazobactam (81.5%).

### K. pneumoniae

We evaluated AMR prediction in 687 *K. pneumoniae* isolates for 13 antimicrobials using non-susceptibility clinical MIC breakpoints (Table 3). A total of 384 isolates were from the CNISP CPE surveillance program, and 303 were non-CPE isolates from the CANWARD surveillance program. The data set was biased toward carbapenemase-producing *K. pneumoniae* since our objective was to validate and improve AMR prediction in CPEs; 50.4% and 54.3% of *K. pneumoniae* were non-susceptible to meropenem based on phenotypic and genotypic data, respectively (Table S1A). The *OqxAB* efflux pump is intrinsic to *K. pneumoniae,* and these genes were not associated with clinical resistance in our data set (29); thus, these two operon genes were removed from our prediction results. Since <1% of *K. pneumoniae* isolates were non-susceptible to colistin or tigecycline, there were insufficient data to assess sensitivities for these two antimicrobials.

**TABLE 3:** *In silico* antimicrobial resistance predictive statistics for *Klebsiella pneumoniae* using Staramr and non-susceptible clinical breakpoints (*n* = 687)

	Non-susceptible		Susceptible		Sensitivity (%)	Specificity (%)	Concordance (%)	ME^*b*^ (%)	VME^*b*^ (%)	CLSI^*c*^ tier of drug
Antimicrobial^*a*^	TP^*b*^	FN^*b*^	TN^*b*^	FP^*b*^						
CEF	430	2	185	70	99.5	72.6	89.5	27.5	0.5	2
CAZ	430	3	196	58	99.3	77.2	91.1	22.8	0.7	4
CRO	317	146	195	29	68.5	87.1	74.5	13.0	31.5	1
CIP	318	82	270	17	79.5	94.1	85.6	5.9	20.5	1
COL	4	21	662	0	NA^*d*^	100	96.9	0.0	84.0	
DOX	148	146	389	4	50.3	99.0	78.2	1.0	49.7	
ERT	335	61	288	3	84.6	99.0	90.7	1.0	15.4	2
GEN	227	11	438	11	95.4	97.6	96.8	2.5	4.6	1
MER	329	17	297	44	95.1	87.1	91.1	12.9	4.9	2
TZP	419	21	228	19	95.2	92.3	94.2	7.7	4.8	1
TGC	0	0	647	40	NA^*d*^	94.2	94.2	5.8	NA^*e*^	
TOB	346	2	326	13	99.4	96.2	97.8	3.8	0.6	2
SXT	357	6	312	12	98.4	96.3	97.4	3.7	1.7	1
Average					87.7	91.7	90.6			

^*a*^ CEF, cefepime; CAZ, ceftazidime; CRO, ceftriaxone; CIP, ciprofloxacin; COL, colistin; DOX, doxycycline; ERT, ertapenem; GEN, gentamicin; MER, meropenem; TZP, piperacillin-tazobactam; TGC, tigecycline; TOB, tobramycin; and SXT, trimethoprim-sulfamethoxazole.

^*b*^ TP, true positive; FN, false negative; TN, true negative; FP, false positive; ME, major error; and VME, very major error.

^*c*^ CLSI, Clinical and Laboratory Standards Institute M100-ED35:2025.

^*d*^ Less than 1% of *K. pneumoniae* isolates were non-susceptible to colistin or tigecycline; therefore, sensitivity could not be assessed for these antimicrobials.

^*e*^ Unable to calculate as true positives and false negatives are equal to 0 (no non-susceptible isolates).

The concordance between phenotypic and genotypic determination of resistance for 13 antimicrobials in *K. pneumoniae* averaged 90.6% (range, 74.5%–97.8%) (Table 3). The sensitivity of prediction of AMR resistance averaged 87.7% (range, 50.3%–99.5%), and the specificity averaged 91.7% (72.6%–100%). Sensitivity was ≥85% for seven antimicrobials (cefepime, ceftazidime, gentamicin, meropenem, piperacillin-tazobactam, tobramycin, and trimethoprim-sulfamethoxazole) and <85% for ertapenem (84.6%), ciprofloxacin (79.5%), ceftriaxone (68.5%), and doxycycline (50.3%). The gene *aac(6″)-Ib-cr* had a change in interpretation in a recent version of the CGE drug-gene key and was missing in false negatives (genotype-susceptible/phenotype-non-susceptible) for ciprofloxacin. All specificities were ≥85%, except for cefepime (72.6%) and ceftazidime (77.2%), suggesting that non-susceptibility was not frequently overcalled by AMR prediction. Concordance for all antimicrobials was ≥85%, except for doxycycline (78.2%) and ceftriaxone (74.5%).

### Methicillin-resistant *Staphylococcus aureus*

We evaluated AMR prediction in 2,150 MRSA isolates for 13 antimicrobials using non-susceptibility clinical MIC breakpoints (Table 4). Tigecycline was evaluated with an FDA cutoff. Staramr detects the *mecA* gene, which was found in all 2,149 isolates from the MRSA surveillance program. The presence of *mecA* was highly correlated with resistance to cefoxitin and oxacillin (2,149/2,150, 99.9%), with the outlier showing characteristics of a borderline oxacillin-resistant *S. aureus* (30). Dalfopristin was used as a proxy to search for quinupristin-dalfopristin in gram-positives, as the Staramr drug-gene key only lists one drug or the other. Since <1% of MRSA isolates were non-susceptible to chloramphenicol, daptomycin, linezolid, quinupristin-dalfopristin, rifampicin, tigecycline, and vancomycin, there were insufficient data to assess sensitivity for these antimicrobials. Additionally, since <1% of MRSA isolates were susceptible to penicillin, specificity could not be assessed.

**TABLE 4:** *In silico* antimicrobial resistance predictive statistics for methicillin-resistant *Staphylococcus aureus* using Staramr and non-susceptible clinical breakpoints (*n* = 2,150)

	Non-susceptible		Susceptible		Sensitivity (%)	Specificity (%)	Concordance (%)	ME^*b*^ (%)	VME^*b*^ (%)	CLSI^*c*^ tier of drug
Antimicrobial^*a*^	TP^*b*^	FN^*b*^	TN^*b*^	FP^*b*^						
CHL	8	620	1,522	0	NA^*d*^	100	71.2	0	98.7	
CIP	1,513	4	617	16	99.7	97.4	99.1	2.5	0.3	4
CLI	799	8	1,321	22	99.0	98.3	98.6	1.6	1.0	1
DAP	0	8	2,142	0	NA^*d*^	100	99.6	0.0	100	2
ERY	1,610	11	509	20	99.3	96.2	98.6	3.8	0.7	1
LZD	0	1	2,149	0	NA^*d*^	100	100	0.0	100	2
PEN	1,943	203	3	1	90.5	NA^*e*^	90.5	25.0	9.5	2
SYN	0	0	2,150	0	NA^*d*^	100	100	0.0	NA^*f*^	
RIF	19	1	2,129	1	NA^*d*^	99.9	99.9	0.1	5.0	3
TET	161	7	1,977	5	95.8	99.7	99.4	0.3	4.2	1
TGC	0	1	2,149	0	NA^*d*^	100	100	0.0	100	
SXT	31	4	2,012	103	88.6	95.1	95.0	4.9	11.4	1
VAN	0	0	2,150	0	NA^*d*^	100	100	0.0	NA^*f*^	1
Average					95.5	98.9	96.3			

^*a*^ CHL, chloramphenicol; CIP, ciprofloxacin; CLI, clindamycin; DAP, daptomycin; ERY, erythromycin; LZD, linezolid; PEN, penicillin; SYN, quinupristin-dalfopristin; RIF, rifampicin; TET, tetracycline; TGC, tigecycline; SXT, trimethoprim-sulfamethoxazole; and VAN, vancomycin.

^*b*^ TP, true positive; FN, false negative; TN, true negative; FP, false positive; ME, major error; and VME, very major error.

^*c*^ CLSI, Clinical and Laboratory Standards Institute M100-ED35:2025.

^*d*^ Less than 1% of MRSA isolates were non-susceptible to these antibiotics; therefore, sensitivity could not be assessed.

^*e*^ Less than 1% of MRSA isolates were susceptible to these antibiotics; therefore, specificity could not be assessed.

^*f*^ Unable to calculate as true positives and false negatives are equal to 0 (no non-susceptible isolates).

The concordance between broth microdilution and AMR prediction of resistance for 13 antimicrobials in MRSA averaged 96.3% (range, 71.2%–100%) (Table 4). The sensitivity of prediction of AMR resistance averaged 95.5% (range, 88.6%–99.7%), and the specificity averaged 98.9% (range, 95.1%–100%). Sensitivities were ≥85% for all antimicrobials that could be assessed. Specificities were ≥85% for all antimicrobials that were assessed, suggesting that non-susceptibility was not frequently overcalled by AMR prediction. Concordances for all antimicrobials were ≥85%, except for chloramphenicol (71.2%).

### Vancomycin-resistant *Enterococcus faecium*

We evaluated AMR prediction in 944 VRE isolates for 10 antimicrobials using non-susceptibility clinical MIC breakpoints (Table 5). Tigecycline was evaluated with an EUCAST cutoff. Of the 922 isolates that were non-susceptible to vancomycin phenotypically, 917 had a *van* gene, with 885 having *vanHAX* (96.5%) and 32 with *vanHBX* (3.5%). The remaining five isolates (0.5%) were false negatives (genotype-susceptible/phenotype-non-susceptible) but were found to have the *vanA* gene through PCR testing, which may be due to gaps in DNA completeness or sequencing coverage. Since <1% of VRE isolates were non-susceptible to daptomycin, linezolid, and tigecycline, there were insufficient data to calculate sensitivity for these antimicrobials. Conversely, <1% of VRE isolates were susceptible to ampicillin, ciprofloxacin, erythromycin, penicillin, and vancomycin, and there were insufficient data to assess specificity for these antimicrobials.

**TABLE 5:** *In silico* antimicrobial resistance predictive statistics for vancomycin-resistant *Enterococcus faecium* using Staramr and non-susceptible clinical breakpoints (*n* = 944)

	Non-susceptible		Susceptible		Sensitivity (%)	Specificity (%)	Concordance (%)	ME^*b*^ (%)	VME^*b*^ (%)	CLSI^*c*^ tier of drug
Antimicrobial^*a*^	TP^*b*^	FN^*b*^	TN^*b*^	FP^*b*^						
AMP	937	4	2	1	99.6	NA^*d*^	99.5	33.3	0.4	1
CHL	239	90	608	7	72.6	98.9	89.7	1.1	27.4	
CIP	941	1	1	1	99.9	NA^*d*^	99.8	50.0	0.1	2 (urine)
DAP	0	38	906	0	NA^*e*^	100	96.0	0.0	100	2
ERY	923	0	0	21	100	NA^*d*^	97.8	100	0.0	
LZD	2	26	915	1	NA^*e*^	99.9	97.1	0.1	92.9	2
PEN	938	4	2	0	99.6	NA^*d*^	99.6	0.0	0.4	1
TET	660	25	235	24	96.4	90.7	94.8	9.3	3.7	3 (urine)
TGC	0	1	943	0	NA^*e*^	100	99.9	0.0	100	
VAN	922	5	5	12	99.5	NA^*d*^	98.2	70.6	0.5	2
Average					95.4	97.9	97.2			

^*a*^ AMP, ampicillin; CHL, chloramphenicol; CIP, ciprofloxacin; DAP, daptomycin; ERY, erythromycin; LZD, linezolid; PEN, penicillin; TET, tetracycline; TGC, tigecycline; and VAN, vancomycin.

^*b*^ TP, true positive; FN, false negative; TN, true negative; FP, false positive; ME, major error; and VME, very major error.

^*c*^ CLSI, Clinical and Laboratory Standards Institute M100-ED35:2025.

^*d*^ Less than 1% of VRE isolates were susceptible to these antibiotics; therefore, specificity could not be assessed.

^*e*^ Less than 1% of VRE isolates were non-susceptible to these antibiotics; therefore, sensitivity could not be assessed.

The concordance between broth microdilution and AMR prediction of resistance for 10 antimicrobials in VRE averaged 97.2% (range, 89.7%–99.9%) (Table 5). Sensitivity of prediction of AMR resistance averaged 95.4% (range, 72.6%–100%), while specificity averaged 97.9% (90.7%–100%). Sensitivities were ≥85% for all antimicrobials that could be assessed, except for chloramphenicol (72.6%). Specificities were ≥85% for all antimicrobials that could be assessed. Concordances for all antimicrobials were ≥85%. Of note, we removed two additional antimicrobials due to very low concordance values: quinupristin-dalfopristin (77.3% and sensitivity 10.2%) and rifampicin (4.8%).

## DISCUSSION

As of 2019, more than 46 AMR bioinformatics tools were documented, and these vary in terms of input/outputs, databases used, and how AMR gene searches are performed (31). While each tool has its strengths and limitations, all tools are subject to the limitations of the specificity, complexity, and completeness of the genes and mutation databases used. Based on a review of various AMR prediction programs, high concordance was observed for several bacterial species when comparing phenotypic and genotypic AMR detection (31). In this review, studies that investigated MRSA, VRE, and CPEs interpreted MIC values based on clinical breakpoints or an epidemiological cutoff value, and all sensitivity and specificity values were >87% while concordances were >96.5%. Within these studies, several AMR prediction software tools were used, and sample sizes varied greatly from 24 to 501 isolates per study. As there are many different AMR prediction methodologies to choose from, in general, public health laboratories around the globe employ various AMR methodologies best suited to their analyses (31), but other similar and commonly used AMR prediction tools besides Staramr include the ResFinder web tool, CARD, ARG-ANNOT, AMRFinderPlus, and PATRIC (27, 28, 32, 33). While CARD is highly comprehensive, the database has not yet been correlated with clinical resistance breakpoints, so PHAC cannot use it yet for public health surveillance.

For foodborne pathogen *Salmonella enterica*, PHAC transitioned from traditional antimicrobial susceptibility testing by broth microdilution and the Clinical and Laboratory Standards Institute breakpoints to whole-genome sequencing-based AMR prediction using Staramr (11). WGS-based testing enables characterization of molecular mechanisms, genetic context, and new gene variants, which enables more precise tracking of the spread of resistance genes. WGS is cost-neutral when it is replacing both antimicrobial susceptibility testing and traditional strain typing. The major disadvantage of all *in silico* prediction tools is that they cannot detect resistance genes and mutations that are not in the databases; thus, PHAC continues to test 10% of *Salmonella* isolates by phenotypic broth microdilution to detect emerging resistance and for continual improvements to Staramr.

In this internal tool refinement of Staramr for healthcare-associated pathogens, AMR prediction using Staramr performed well for most combinations of antimicrobials and bacterial species. The average sensitivities (87.7%–95.5%), specificities (90.1%–98.9%), and concordances (90.6%–97.2%) of categorical AMR prediction compared to broth microdilution in this study suggest that AMR prediction is a promising tool for *E. coli, K. pneumoniae*, MRSA, and VRE. Staramr usage in the scientific literature has been increasing for *in silico* AMR prediction, such as in a large-scale study of *S. aureus* that sampled 29,679 publicly available genomes (34). Evaluation of AMR prediction tools is important, as false negatives can cause missed non-susceptibility leading to ineffective treatments, while false positives can mean eliminating a potentially useful treatment (35).

False positives occur when an isolate is determined to be non-susceptible by the WGS-based test but not the MIC test. This usually occurs when the isolate carries a known resistance gene that produces an elevated MIC that is just below the breakpoint of non-susceptibility. For example, cefepime and ceftazidime were discordant in both *E. coli* and *K. pneumoniae* with specificities of 70.7%–77.2% (Tables 2 and 3). In the discordant pair, cefepime and *E. coli*, the genes *bla_OXA-1_*, *bla_CTX-M-14/15/27_*, and *bla_KPC-2_* predicted non-susceptibility but often produced elevated MICs that were just below the non-susceptible breakpoint. The genes *bla_CTX-M-14/15/27_* also produced elevated MICs below the breakpoint in *E. coli* and ceftazidime. For *K. pneumoniae* and cefepime, the correlation of genes producing elevated MICs below the breakpoint was not as strong, but the genes *bla_OXA-181_*, *bla_CTX-M-14/15/27_*, *bla_KPC-2/3_*, and several *bla_SHV_* variants were observed throughout all MIC categories. These same genes, except for *bla_OXA-181_*, also resulted in discordances for *K. pneumoniae* and ceftazidime. Additionally, for *E. coli* and ciprofloxacin (specificity, 74.9%), lower specificity was due to *gyrA* S83L and *parE* I529L producing a range of MICs, and for piperacillin-tazobactam (specificity, 79.6%), *bla_OXA-1_* was seen throughout all MIC categories, causing decreased specificity. Interestingly, MIC testing is only accurate within one to two twofold dilutions; therefore, MIC results that are close to the breakpoint may result in categorization errors (36).

Lower sensitivity is caused by false negatives (WGS susceptible/non-susceptible MIC), usually due to incomplete databases and unknown mechanisms (11). To address false negatives, we cross-checked these isolates against other AMR prediction software such as CARD and AMRFinderPlus. This identified additional genes, mutations, and drug-gene interpretations which will be added to future updates of Staramr because they were supported by elevated MICs in our data set. For example, for the discordant organism-antimicrobial pair, colistin and *E. coli*, detection of colistin resistance can be greatly improved by adding additional genetic markers to the Staramr gene-drug key. Future versions of Staramr will incorporate *pmrB* alleles (E123D, P94S, and C84Y), which is also supported by the literature (37–39). This would change all false-negative isolates to true positives, which would increase the sensitivity to 100%.

In our data set, the *E. coli* isolates with these *pmrB* alleles had MICs of >8 and 4 mg/L, indicating increased phenotypic non-susceptibility to colistin. In *K. pneumoniae* isolates, there were a high number of false negatives for colistin. Given the lack of knowledge of chromosomal mutations, prediction of colistin non-susceptibility in *K. pneumoniae* is limited to acquired *mcr* (mobilizable colistin resistance) genes (15, 40). For determination of colistin resistance in *K. pneumoniae*, phenotypic antimicrobial susceptibility should be performed. Additionally, we identified that the lower sensitivity to ceftriaxone was due to several *bla* genes in the drug-gene key coding for cefotaxime but not ceftriaxone (both are third generation cephalosporins). Adding ceftriaxone to the phenotypic profile of several *bla* genes and their associated alleles in a future update of Staramr, namely *bla*_CMY_, *bla*_KPC_, *bla*_NDM_, *bla*_ACT_, and *bla*_FOX_, would improve the sensitivity of prediction of ceftriaxone resistance to 98.0% in *E. coli* and 98.9% in *K. pneumoniae*. Specificity would remain similar, going from 97.9% to 97.6% for *E. coli* and 87.1% to 86.6% for *K. pneumoniae*. Generally, prediction of resistance was slightly better for *E. coli* compared to *K. pneumoniae*; potential reasons for this include the poorly defined contribution of outer membrane porins that are found in *K. pneumoniae* and potentially other undiscovered resistance determinants (41, 42). Potential reasons for low ertapenem sensitivity in *E. coli* could be outer membrane porin mutations and overexpression of AmpC β-lactamase (43, 44).

There are limitations to our internal tool refinement. The first being that CLSI breakpoints are continuously updated, which would cause the MIC threshold to change. These findings of the correlation between genotype and phenotype may inform future CLSI and/or EUCAST breakpoint revisions. Second, the data set was biased toward CPEs, MRSA, and VRE since our objective was to validate Staramr with these priority organisms. This was partially mitigated by including data from susceptible isolates from CANWARD. For some organism-antimicrobial combinations, sensitivity, specificity, or very major error could not be assessed because there were too few non-susceptible or susceptible isolates, respectively, for that antimicrobial. The data set also lacked non-CP carbapenem-resistant *Enterobacterales* and non*-faecium Enterococcus* species. Finally, as with all AMR prediction tools, a limitation with some antimicrobials is the current lack of knowledge of all genetic factors that contribute to resistance, including promoter mutations, porin mutations, and efflux pumps. Due to limited knowledge of the mechanisms of resistance and potentially additive effects between multiple mechanisms, phenotypic non-susceptibility correlated very poorly with a single gene or mutation for some antimicrobial-organism combinations such as VRE with rifampicin and quinupristin-dalfopristin. Rifampicin *in silico* resistance prediction in VRE also proved unreliable in another AMR prediction software study, demonstrating that this organism-antimicrobial combination needs further exploration of genetic mechanisms of resistance (45). Additionally, dalfopristin was a better predictor of phenotypic quinupristin-dalfopristin resistance in MRSA, and this presents an opportunity to refine the gene-drug key.

*In silico* AMR prediction presents challenges but also clear benefits, particularly as resistance rates continue to rise. Based on our internal tool refinement, *E. coli* and *K. pneumoniae* AMR prediction was generally strong, though drug-gene key and threshold adjustments may be warranted for some antimicrobials for optimal AMR prediction. Predictions for MRSA and VRE were also robust. This paper identified areas for refinement in both ResFinder and PointFinder used for AMR prediction. Looking forward, a helpful resource that is in progress will be The European Society of Clinical Microbiology and Infectious Diseases Study Group for Epidemiological Markers and EUCAST’s guidelines for organism-specific AMR prediction and ontology rules for assigning levels of resistance (46). Overall, this study demonstrates that WGS-based AMR prediction using Staramr may be a promising approach for rapid resistance detection in the priority healthcare-associated pathogens, *E. coli*, *K. pneumoniae*, MRSA, and VRE. Due to low performance for specific combinations of antimicrobials and pathogens, continued refinement of databases, thresholds, and interpretive rules will further improve reliability and utility in public health and clinical microbiology.

## Data Availability

Sequence read data for isolates were submitted to the National Center for Biotechnology Information (NCBI) Sequence Read Archive (SRA) under BioProject accession numbers PRJNA1515817 (VRE from CNISP), PRJNA1522379 (MRSA from CNISP), PRJNA855907 (CPE from CNISP), PRJNA1526765 (*E. coli* from CANWARD), and PRJNA1524002 (*K. pneumoniae* from CANWARD). R scripts for analyses can be made available upon request. Staramr outputs for each organism are provided in Tables S2 to S5.

## SUPPLEMENTAL MATERIAL

Supplemental figure and table spectrum.01652-26-s0001.pdf Figure S1 and Tables S1A and B.

TABLE S2 spectrum.01652-26-s0002.xlsx Staramr antimicrobial resistance prediction results from *Escherichia coli* isolates (n = 1095).

Table S3 spectrum.01652-26-s0003.xlsx Staramr antimicrobial resistance prediction results from *Klebsiella pneumoniae* isolates (n = 687).

Table S4 spectrum.01652-26-s0004.xlsx Staramr antimicrobial resistance prediction results from MRSA isolates (n = 2150).

Table S5 spectrum.01652-26-s0005.xlsx Staramr antimicrobial resistance prediction results from VRE isolates (n = 944).

ASM does not own the copyrights to Supplemental Material that may be linked to, or accessed through, an article. The authors have granted ASM a non-exclusive, world-wide license to publish the Supplemental Material files. Please contact the corresponding author directly for reuse.
