# Supplementary material for: Genomic prediction of antimicrobial resistance in priority healthcare pathogens using Staramr
Source: Microbiol Spectr. 2026 Oct 6;14(11):e01652-26. doi: 10.1128/spectrum.01652-26 (PMC13729218; doi:10.1128/spectrum.01652-26)
Supplement: Supplementary file 1 [file spectrum.01652-26-s0001.pdf]

Supplementary Figure

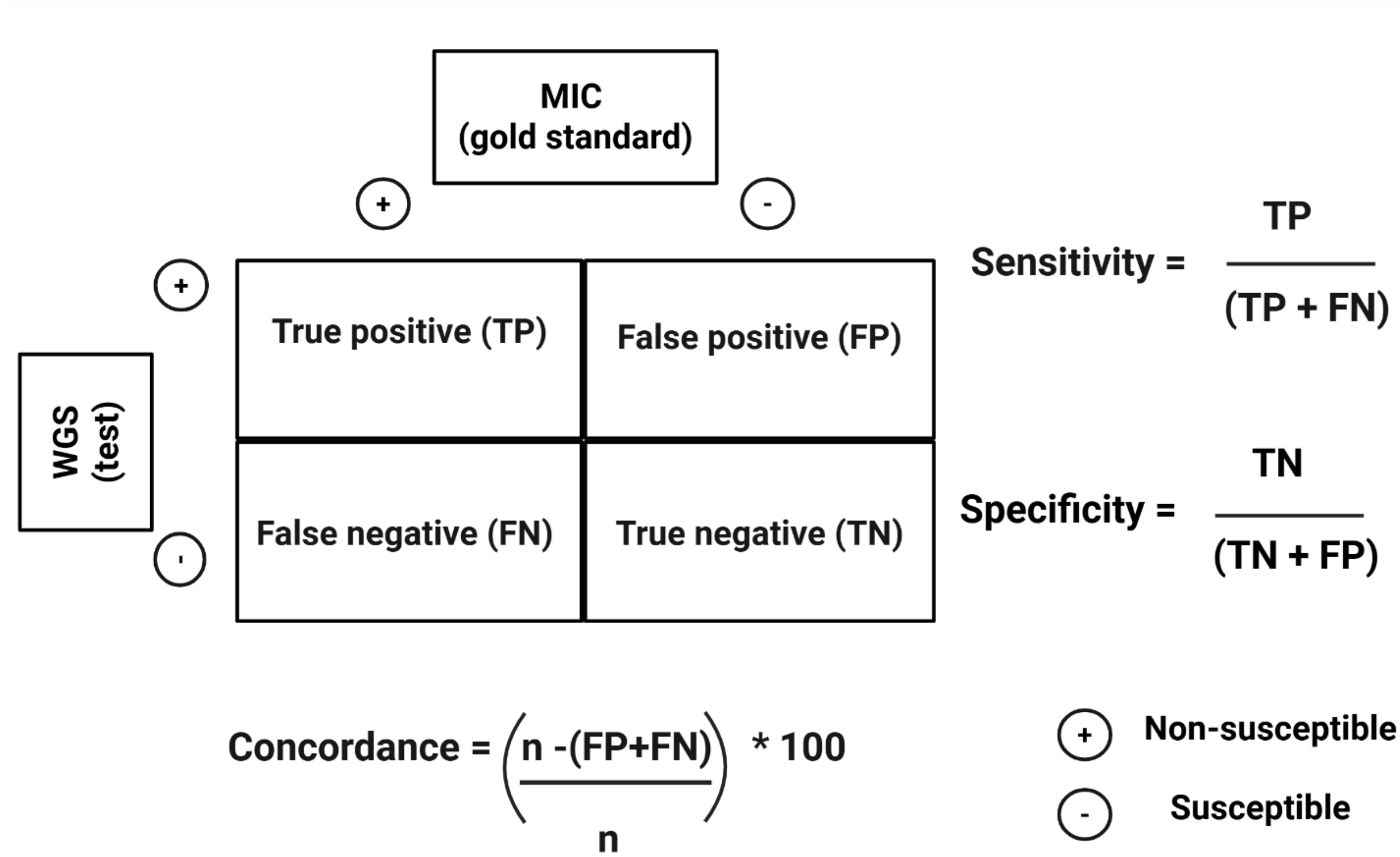

Supplementary Figure 1. Confusion matrix visualization and calculations for predictive statistics. MIC: Minimum inhibitory concentration, WGS: Whole-genome sequencing. Image created in BioRender.com.

Supplementary Tables

Table 1A. Proportion of non-susceptible isolates in Gram-negative organisms in this dataset, which was biased for carbapenemase producers.

Antimicrobial	% of non-susceptible *E. coli* by broth microdilution[1]	% of non-susceptible *E. coli* by whole-genome sequencing	% of non-susceptible *K. pneumoniae* by broth microdilution[1]	% of non-susceptible *K. pneumoniae* by whole-genome sequencing
**Cefepime**	56.3	67.1	62.9	72.8
**Ceftazidime**	56.7	68.6	63.0	71.0
**Ceftriaxone**	68.9	57.9	67.4	50.4
**Ciprofloxacin**	68.7	76.3	58.2	48.8
**Colistin**	0.7	0.3	3.6	0.6
**Doxycycline**	55.8	55.4	42.8	22.1
**Ertapenem**	26.4	18.5	57.6	49.2
**Gentamicin**	36.0	35.6	34.6	34.6
**Meropenem**	17.2	20.1	50.4	54.3
**Piperacillin-tazobactam**	36.8	44.0	64.0	63.8
**Tigecycline**	0.0	0.1	2.3	5.8
**Tobramycin**	45.3	46.6	50.7	52.3
**Trimethoprim-sulfamethoxazole**	58.7	58.9	52.8	53.7

1. Non-susceptible as defined by non-susceptible clinical MIC breakpoints detailed in Table 1. Tigecycline is based on FDA cutoffs.

Table 1B. Proportion of non-susceptible isolates in the Gram-positive dataset containing MRSA and VRE.

Antimicrobial	% of non-susceptible MRSA by broth microdilution[1]	% of non-susceptible MRSA by whole-genome sequencing	% of non-susceptible VRE by broth microdilution[1]	% of non-susceptible VRE by whole-genome sequencing
**Ampicillin**	NA	NA	99.7	99.4
**Chloramphenicol**	29.2	0.4	34.9	26.1
**Ciprofloxacin**	70.6	71.1	99.8	99.8
**Clindamycin**	37.5	38.2	NA	NA
**Daptomycin**	0.4	0.0	4.0	0.0
**Erythromycin**	75.4	75.8	97.8	100
**Linezolid**	0.0	0.0	3.0	0.3
**Penicillin**	99.8	90.4	99.8	99.4
**Quinupristin-dalfopristin**	0.0	0.0	NA	NA
**Rifampicin**	0.9	0.9	NA	NA
**Tetracycline**	7.8	7.7	72.6	72.5
**Tigecycline**	0.0	0.0	0.1	0.0
**Trimethoprim-sulfamethoxazole**	1.6	6.2	NA	NA
**Vancomycin**	0.0	0.0	98.2	98.9

1. Non-susceptible as defined by non-susceptible clinical MIC breakpoints detailed in Table 1. Tigecycline is based on FDA cutoffs for MRSA and EUCAST cutoffs for VRE. Clindamycin has in-house broth microdilution interpretation criteria.

Supplementary tables S2-S5 are present as Excel files.
